# CSCO guidelines for metastatic colorectal cancer: personalized medicine in clinical practice

**DOI:** 10.20892/j.issn.2095-3941.2023.0211

**Published:** 2023-09-09

**Authors:** Mengyuan Yang, Ziheng Xu, Mi Mi, Yuwei Ding, Yuefen Pan, Ying Yuan, Weijing Sun, Shanshan Weng

**Affiliations:** 1 Department of Medical Oncology (Key Laboratory of Cancer Prevention and Intervention, Chinese National Ministry of Education, Key Laboratory of Molecular Biology in Medical Sciences, Zhejiang Province, China), The Second Affiliated Hospital, Zhejiang University School of Medicine, Hangzhou 310009, China; 2 Department of Medical Oncology, Huzhou Central Hospital, Huzhou 313000, China; 3 Zhejiang Provincial Clinical Research Center for Cancer, Hangzhou 310009, China; 4 Cancer Center of Zhejiang University, Hangzhou 310058, China; 5 Binjiang Institute of Zhejiang University, Hangzhou 310052, China; 6 Department of Medical Oncology, The University of Kansas, School of Medicine, Lawrence, KS 66045, United States

Colorectal cancer (CRC) is the second most common cancer in China, the morbidity and mortality rates of which are rapidly increasing^1,2^. Among newly-diagnosed CRC patients, 20% have metastatic disease at the time of presentation and an additional 25% present with localized disease and will subsequently develop metastases^3^. The treatment of metastatic colorectal cancer (mCRC) is gradually moving towards the era of precision therapy, which involves guided treatment based on individual genetic characteristics^4^. Since the first edition of the Chinese Society of Clinical Oncology (CSCO) guidelines was published in 2017, the guidelines have been updated annually according to the latest clinical trial findings^5–9^. Herein we summarize how the CSCO guidelines enable tailor-made treatments of mCRC with different molecular characteristics (**Figure 1**).

**Figure 1:**
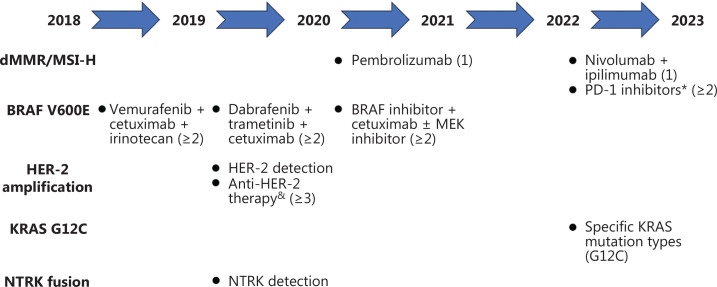
A diagram summarizing CSCO guideline updates. The numbers in brackets represent the number of treatment lines recommended by the corresponding regimen. *Pembrolizumab, envafolimab, serplulimab, tislelizumab, and nivolumab + ipilimumab are preferred. ^&^Trastuzumab + pertuzumab or trastuzumab + lapatinib are recommended.

## Treatment of mismatch repair-deficient (dMMR)/microsatellite instability-high (MSI-H) mCRC

Approximately 10%–15% of CRC patients present with MSI-H/dMMR and have a better prognosis compared to microsatellite stable (MSS)/mismatch repair proficient (pMMR) CRC patients. Thus, the proportion of MSI-H/dMMR patients among all mCRC patients is lower (4%–5%).

KEYNOTE-177 was a landmark study involving immune checkpoint inhibitor (ICI) use in the treatment of mCRC (NCT02563002). A total of 307 patients with MSI-H/dMMR mCRC who had not previously received treatment were randomly assigned to receive either pembrolizumab or chemotherapy (mFOLFOX6 or FOLFIRI with or without bevacizumab or cetuximab). Pembrolizumab treatment resulted in a significantly longer progression-free survival (PFS) than chemotherapy (16.5 *vs*. 8.2 months; *P* = 0.0002), with a considerably lower occurrence of treatment-related grade ≥ 3 adverse events (22% *vs*. 66%). Therefore, pembrolizumab was added as a class I recommendation (Level 1A evidence) in the first-line treatment for MSI-H/dMMR mCRC patients to the 2021 CSCO guidelines^5^.

CheckMate-142 was another important study involving ICIs in the treatment of MSI-H/dMMR mCRC. As early as 2018, the significance of a double-immune treatment strategy (nivolumab + ipilimumab) was affirmed in the late-line treatment of mCRC patients with MSI-H/dMMR (NCT02060188); however, due to the lack of availability of ipilimumab, CSCO guidelines have not recommended this regimen for the palliative treatment of MSI-H/dMMR mCRC patients. The 5-year follow-up results of nivolumab plus low-dose ipilimumab in the first-line therapy cohort from the phase II CheckMate-142 study was presented at the American Society of Clinical Oncology (ASCO) in 2022. Nivolumab + ipilimumab achieved a sustained overall survival (OS) and PFS benefits (48-month OS rate = 72%; 48-month PFS rate = 51%), as well as an encouraging objective response rate (ORR = 71%). Because ipilimumab had been approved in China, this double-ICI treatment regimen was added as a class III recommendation for palliative first-line treatment of metastatic dMMR/MSI-H CRC patients (Level 3 evidence) in the 2023 CSCO guidelines^9^.

PD-1 inhibitors were added as a class II recommendation (Level 2A evidence) for the late-line treatment of MSI-H/dMMR mCRC patients; however, the detailed types of PD-1 inhibitors were not defined in the 2021 CSCO guidelines. Domestic drugs [envafolimab (NCT03667170), tislelizumab (NCT03736889) and serplulimab (NCT03941574)] have shown good efficacy in the treatment of MSI-H/dMMR mCRC patients who had previously failed standard therapy with ORRs of 43.1%, 39.1%, and 46.7%, respectively. Pembrolizumab, envafolimab, serplulimab, tislelizumab, and nivolumab + ipilimumab were recommended as a priority in the late-line treatment of MSI-H/dMMR mCRC patients in the 2023 CSCO guidelines^9^.

Insights: Researchers continue to try improving the cure rate in mCRC patients on the basis of first-line immunotherapy. COMMIT was a phase III clinical study that evaluated the efficacy and safety of atezolizumab monotherapy versus mFOLFOX6 + bevacizumab + atezolizumab as first-line therapy in MSI-H/dMMR mCRC patients (NCT02997228). The COMMIT trial may determine whether the addition of chemotherapy and an anti-angiogenic agent to an ICI further improved the efficacy in MSI-H/dMMR mCRC patients. CheckMate 8HW was another phase III clinical trial that compared the efficacy of three regimens in the first-line treatment of MSI-H/dMMR mCRC [nivolumab monotherapy, nivolumab + ipilimumab, and chemotherapy (NCT04008030)].

Additionally, researchers are currently exploring whether ICI can change the adjuvant treatment of MSI-H/dMMR patients. ATOMIC is an ongoing phase III randomized trial to determine whether the addition of atezolizumab to adjuvant FOLFOX improves disease-free survival (DFS) versus FOLFOX alone in patients with stage III CRC with dMMR (NCT02912559).

## Treatment of mCRC with a *BRAF* V600E mutation

Approximately 10% of CRC patients carry a *BRAF* gene mutation. Among these patients, 90% carry the *BRAF* V600E mutation and 21% of patients with the *BRAF* V600E mutation also have tumors with dMMR/MSI-H. The 5-year survival rate of patients carrying the *BRAF* V600E mutation is significantly less than wild-type patients.

The BRAF inhibitor vemurafenib was approved by the U.S. food & drug administration (FDA) for the treatment of *BRAF*-mutated melanoma in 2011; however, vemurafenib monotherapy did not provide benefits in patients with *BRAF*-mutated mCRC^10^. Yang et al.^11^ constructed CRC cell lines and corresponding transplanted tumor mouse models carrying the *BRAF* V600E mutation and reported that dual inhibition of BRAF and EGFR by vemurafenib and cetuximab combined with irinotecan (VIC) yielded a significant anti-tumor effect. Furthermore, a phase II clinical trial (SWOG1406) revealed that the VIC regimen resulted in a prolongation of PFS (4.4 *vs*. 2.0 months) and a higher disease control rate (67% *vs*. 22%) than irinotecan plus cetuximab (NCT02164916). Therefore, the VIC regimen was added as a class III recommendation (Level 2B evidence) in the late-line palliative treatment of mCRC patients with the *BRAF* V600E mutation in the 2019 CSCO guidelines^6^.

The mechanism underlying the lower effectiveness of BRAF inhibitor monotherapy treatment in *BRAF*-mutant mCRC compared to melanoma likely involves reactivation of the MAPK pathway downstream of BRAF, which may be overcome by blocking BRAF, EGFR, and MEK together. BEACON CRC was a phase III clinical trial designed to evaluate the efficacy and safety of cetuximab and encorafenib (a selective BRAF kinase inhibitor) with or without binimetinib (a MEK inhibitor) compared to chemotherapy for previously treated mCRC patients with *BRAF* V600E (NCT02928224). The enrolled participants were divided into three groups: triplet-combination (cetuximab + encorafenib + binimetinib); doublet-combination (cetuximab + encorafenib); and the chemotherapy control. The median OS of patients receiving 3-drug treatment was nearly 2-fold compared to the control group and the risk of disease death was reduced by 48%. In addition, data from several other phase I/II clinical trials confirmed the efficacy of dabrafenib + trametinib^12^ and dabrafenib + trametinib + panitumumab (NCT01750918). Therefore, considering drug availability, dabrafenib + trametinib + cetuximab was added as a class III recommendation (Level 2B evidence) in the late-line treatment for patients with *BRAF* V600E in the 2020 CSCO guidelines^7^.

The updated results of the BEACON CRC study at the 2020 ASCO annual meeting showed that the triplet- and doublet-combination groups both achieved a significantly longer OS (9.3 *vs*. 9.3 *vs*. 5.9 months), a longer PFS (4.5 *vs*. 4.3 *vs*. 1.5 months), and a higher ORR (27% *vs*. 20% *vs*. 2%) compared with the control group. Because there was no difference in OS and PFS between the triplet- and doublet-combination groups, the 2021 version of the CSCO guidelines revised the original dabrafenib + trametinib + cetuximab regimen to a BRAF inhibitor + cetuximab ± MEK inhibitor regimen as a class III recommendation (Level 2B evidence)^5^. In addition, a new note was added in the 2021 CSCO guidelines, as follows: “BRAF inhibitor + cetuximab + MEK inhibitor can be considered for patients with extensive metastatic sites, high tumor burden or obvious tumor-related symptoms”^5^.

Insights: The doublet-combination group has also been tested in the first-line setting. BREAKWATER was a phase III clinical trial involving a BRAF inhibitor + cetuximab versus a BRAF inhibitor + cetuximab ± chemotherapy (FOLFOX6 or FOLFIRI) versus two- or three-drug chemotherapy ± bevacizumab in the first-line treatment of mCRC with *BRAF* V600E and MSS. The efficacy data of the safety lead-in period was reported (NCT04607421) at the 2023 ASCO-GI annual meeting. First-line treatment with encorafenib + cetuximab combined with FOLFOX6 or FOLFIRI achieved an encouraging ORR (68.4% and 75.0%, respectively), as well as a promising mPFS (11.1 months and not estimable, respectively). Due to the better efficacy data of FOLFIRI + encorafenib + cetuximab, the study supplemented cohort 3 to compare the efficacy of encorafenib + cetuximab + FOLFIRI (arm D) and FOLFIRI ± bevacizumab (arm E) based on the previous 3 arms (A: encorafenib + cetuximab, B: encorafenib + cetuximab + FOLFOX, and C: FOLFOX/FOLFIRINOX/XELOX ± bevacizumab).

At the same time, investigators have begun to consider whether BRAF inhibitors further improve the DFS after adjuvant therapy in patients with *BRAF*-mutant CRC. The study protocol of a randomized trial (NCT05710406) was recently presented at the 2023 ASCO annual meeting. The protocol was designed to determine if encorafenib + cetuximab improves the DFS in patients with resected *BRAF* V600E and MSS/pMMR high-risk stage II (pT4) or stage III colon cancer after standard adjuvant therapy.

## Treatment of mCRC with *HER-2* amplification

*HER-2*, also known as *ERBB2*, is a member of the *ERBB* family. An abstract presented at the 2017 ASCO annual meeting reported *HER-2* alterations in mCRC patients. Comprehensive genomic sequencing in 8874 mCRC patients was analyzed^13^. A total of 433 (4.9%) *HER-2* alterations were detected, of which 265 (3.0%) were *HER-2* amplifications, 164 (1.9%) were *HER-2* mutations, and 4 (0.5%) were *HER-2* fusions.

In the Molecular Pathological Section of the 2020 CSCO guidelines^7^ “detection of HER-2 status” was added as a class III recommendation for surgery/biopsy specimens of mCRC after standard treatment failure. The HER-2 status detection method was similar to breast and gastric cancers, including immunohistochemistry (IHC) and fluorescence *in situ* hybridization (FISH). Currently, the criteria for judging HER-2 positivity in CRC are only derived from clinical research. The definition of HER-2*-*positive by IHC was as follows in the HERACLES study: ≥ 50% of the tumor tissue was 3+ positive; and for an HER-2 score of 2+, the HER-2 status should be further clarified by FISH (≥ 50% of tumor cells with a HER-2/CEP17 ratio ≥ 2.0).

The HERACLES trial showed that *KRAS* codon 12/13 wild-type, HER-2-positive mCRC patients receiving trastuzumab + lapatinib had a 30% ORR and the PFS reached 21 weeks^14^. Moreover, 57 *HER-2*-amplified mCRC patients enrolled in the MyPathway basket study were treated with pertuzumab + trastuzumab as third-line therapy (NCT02091141). Eighteen patients (32%) achieved an objective response, including one complete response. The median PFS and OS were 2.9 and 11.5 months, respectively. Although there is still a lack of data on anti-HER-2 targeted therapy for *HER-2*-amplified mCRC in the Chinese population, referring to the latest version of the NCCN guidelines, trastuzumab + pertuzumab or trastuzumab + lapatinib is recommended as third-line treatment for *HER2*-amplified mCRC as a class III recommendation (Level 2B evidence) per the 2020 CSCO guidelines^7^.

Insights: To date, the efficacy and survival data of a number of phase I/II clinical trials on the combination of anti-HER-2 monoclonal antibodies and small molecule tyrosine kinase inhibitors in the treatment of *HER-2*-amplified mCRC have been published. MOUTAINEER reported that among HER-2-positive patients, the response rate of tucatinib monotherapy was only approximately 3%, while the response rate of tucatinib + trastuzumab reached 38.1% (NCT03043313). The ongoing MOUNTAINEER-03 study aims to compare the efficacy of tucatinib + trastuzumab + mFOLFOX6 versus mFOLFOX6 + bevacizumab/cetuximab in the first-line treatment of HER-2-positive and *RAS* wild-type mCRC patients (NCT05253651).

The antibody-drug conjugate, trastuzumab deruxtecan (DS-8201), is another attractive HER2-targeted drug. DESTINY-CRC01 demonstrated the efficacy and safety of DS-8201 (6.4 mg/kg) in 53 patients with HER2-positive mCRC (IHC3+ or IHC 2+/FISH+) who failed multiple lines of therapy. The ORR reached 45.3%, but none of the patients responded in the low-level HER2 expression (IHC2+/FISH− or IHC1+) mCRC group (NCT03384940). Interestingly, DESTINY-CRC02 demonstrated that the efficacy-to-risk ratio for DS-8201 at 5.4 mg/kg was better than 6.4 mg/kg, with a higher ORR (37.8% *vs*. 27.5%) and a lower incidence of grade ≥ 3 adverse events (49.4% *vs*. 59.9%) in the treatment of HER-2-positive mCRC patients (IHC3+ or IHC 2+/FISH+) (NCT04744831).

## Treatment of mCRC with a *KRAS* G12C mutation

Although KRAS was described in CRC 30–40 years ago, KRAS was considered to be an undruggable target until the advent of sotorasib (AMG510), which is active against solid tumors with *KRAS* G12C^15^. However, among CRC patients, those patients with *KRAS* G12C account for only 3%–4%^16^. Detection of *KRAS* mutations is a routine clinical practice before treating mCRC. With respect to *KRAS* G12C, the 2023 CSCO guidelines^9^ specifically emphasize that in addition to exons 2, 3, and 4, attention should also be paid to whether the detection method covers some important gene mutation sites and mutation forms, such as G12C and G12D.

Insights: The currently available KRAS G12C inhibitors include sotorasib (AMG510) and adagrasib (MRTX 849). When using a single drug, the effective rate of the two drugs is < 20%. However, if KRAS G12C inhibitors are combined with anti-EGFR therapy, the curative effect is doubled^17,18^. Therefore, the current focus is mainly on the treatment of small-molecule drugs targeting KRAS G12C combined with anti-EGFR therapy. CodeBreak 101 showed that the sotorasib + panitumumab + FOLFIRI regimen was associated with good clinical benefits in the late-line treatment of *KRAS* G12C-mutant mCRC patients, with an ORR of 55% and a disease control rate (DCR) of 93%. The efficacy was not related to the number of previous treatment lines and whether or not irinotecan treatment failed (NCT04185883). Some domestic small-molecule KRAS G12C inhibitors are also worthy of our attention. Both IBI351 [GFH925 (600 mg BID, NCT05005234 and NCT05497336)] and D-1553 (NCT04585035) monotherapy in the treatment of *KRAS* G12C-mutated mCRC have shown great efficacy, with an ORR reaching 42.9% and 20.8%, and a DCR reaching 88.1% and 95.8%, respectively. CodeBreak 300 is an ongoing study comparing sotorasib + panitumumab with standard third-line therapy (NCT05198934). The KRYSTAL-10 study, which compares adagrasib + cetuximab head-to-head with standard second-line therapy (NCT04793958), is similar and also ongoing.

## Treatment of mCRC with *NTRK* gene fusion

*NTRK* gene fusion is very rare in CRC, with an incidence of approximately 0.35%. In the basket study of NTRK inhibitors treating *NTRK* gene fusion tumors, mCRC with *NTRK* gene fusions showed a gratifying response rate to NTRK inhibitors; 4 patients with mCRC who failed multiple lines of therapy received larotrectinib (NCT02637687 and NCT02576431), with an ORR of 75%, and 4 patients received entrectinib with (NCT02097810, NCT02568267 and EudraCT 2012-000148-88), an ORR of 50%.

Like HER-2 status detection, the 2020 CSCO guidelines also added “detection of *NTRK* gene fusion” as a class III recommendation for surgery/biopsy specimens of mCRC after standard treatment failure^7^. There are several methods for detecting *NTRK* gene fusion, including IHC, FISH, DNA-based next-generation sequencing (NGS), and RNA-based NGS^19^. IHC is a fast and economical method for primary screening with a sensitivity of 87.9% and a specificity of 81.1%, but *NTRK* gene fusion still needs to be verified by DNA-based NGS. Moreover, RNA-based NGS is considered to be the best detection method for *NTRK* gene fusion.

## Conclusions and insights

This editorial mainly focused on the research pertaining to the treatment of mCRC with relatively rare molecular features, such as dMMR/MSI-H, *BRAF* V600E, *HER-2* amplification, *KRAS* G12C, and *NTRK* gene fusion. In addition to these alterations, some molecular characteristics are also worthy of our attention, such as the *PIK3CA* mutation, *MET* amplification, *POLE/POLD1* mutation, *ALK* gene fusion, and CLDN18.2 expression. Compared to the international guidelines, the CSCO guidelines focus more on the results of clinical trials based on the Chinese population and recommend appropriate treatment options considering the availability of drugs in China. It is believed that with the increasingly intense international information exchange, the CSCO guidelines will continue to approach the latest anti-tumor drugs, while maintaining unique Chinese characteristics.
